# The St. Louis Medical and Surgical Journal

**Published:** 1872-01

**Authors:** 


THE ST. LOUIS MEDICAL AND SURGICAL JOURNAL.—This valuable medical journal will, in the future, be published monthly, instead of bi-monthly as heretofore. The new editors are Drs. William S. Edgar and H. Z. Gill.

